# Wip1 Deficiency Promotes Neutrophil Recruitment to the Infection Site and Improves Sepsis Outcome

**DOI:** 10.3389/fimmu.2017.01023

**Published:** 2017-08-22

**Authors:** Xiao-Fei Shen, Yang Zhao, Ke Cao, Wen-Xian Guan, Xue Li, Qian Zhang, Yong Zhao, Yi-Tao Ding, Jun-Feng Du

**Affiliations:** ^1^Department of General Surgery, Nanjing Drum Tower Hospital, The Affiliated Hospital of Nanjing University Medical School, Nanjing, China; ^2^Transplantation Biology Research Division, State Key Laboratory of Membrane Biology, Institute of Zoology, Chinese Academy of Sciences, Beijing, China; ^3^Department of Critical Care Medicine, Nanjing Drum Tower Hospital, The Affiliated Hospital of Nanjing University Medical School, Nanjing, China; ^4^Department of General Surgery, PLA Army General Hospital, Beijing, China

**Keywords:** wild-type p53-induced phosphatase 1, neutrophil migration, cecal ligation and puncture, CXC chemokine receptor 2, sepsis, septic shock

## Abstract

Sepsis is defined as an uncontrolled host response to infection, and no specific therapy or drugs have been used in clinical trials currently. Discovering new therapeutic targets for sepsis treatment has always been a central problem in the field of sepsis research. Neutrophils stand at the first line in controlling infection and have been identified to be dysregulated with impaired migration and antimicrobial function during sepsis. Based on our previous results on demonstrating wild-type p53-induced phosphatase 1 in controlling neutrophil development, we explored the possible relationship among Wip1, neutrophils, and sepsis in the present study. Wip1-deficient mice exhibited improved outcomes in cecal ligation and puncture (CLP)-induced sepsis model with enhanced bacterial clearance and less multi-organ damage. The protection seen in Wip1 KO mice was mainly due to an increased accumulation of neutrophils in the primary infectious locus mediated by the decreased internalization of CXCR2, as well as by an increased antimicrobial function. Additionally, we also identified a negative correlation between CXCR2 and Wip1 in human neutrophils during sepsis. Pharmacological inhibition of Wip1 with its inhibitor can also prevent the internalization of CXCR2 on human neutrophils treated with lipopolysaccharides *in vitro* and significantly improve the outcome in CLP-induced sepsis model. Taken together, our results demonstrate that Wip1 can negatively regulate neutrophil migration and antimicrobial immunity during sepsis and inhibition of Wip1 can be a potential therapeutic target for sepsis treatment.

## Introduction

Sepsis is defined as a dysregulated host response to infection (1, 2) and is still a major cause of death in hospitalized patients. Development of novel therapeutics, which can be specific for sepsis treatment remains to be discovered. The difficulty in finding novel therapeutic strategies for sepsis is the complicated pathophysiology process, which can be defined as an interplay between host pro-inflammatory and anti-inflammatory responses (3). Recent evidence showed that most patients with sepsis survived the hyper-inflammatory phase, but failed to control the primary infection and/or the acquisition of secondary hospital-acquired infection due to the altered immune function (4). Both innate and adaptive immune responses have been altered after such “recovery” from acute events with a predominant immunosuppression status (5). Thus, efforts focused on reversing the immune cell dysfunction may be a promising perspective in the area of sepsis research.

Wild-type (WT) p53-induced phosphatase 1 (Wip1) belongs to the PP2C family and is involved in numerous physiology and pathophysiology process (6). Previous studies showed that Wip1 participated in stress-induced networks and tumor development (7), and recent studies also suggested the critical role of Wip1 in aging (8) and neurogenesis (9). Furthermore, the role of Wip1 in immunity has also been discovered. Wip1 can regulate the development and/or function of both the innate and adaptive immune cells including neutrophils, macrophages, T cells, and B cells (6) and controls immune response through the interplay with inflammatory signaling pathways such as NF-κB and p38 MAPK as well (3). Based on these results, Wip1 has been suggested to be promising therapeutic targets in inflammatory bowel diseases (10) and intestinal ischemia/reperfusion injury (11, 12). However, whether Wip1 participated in systemic immune response and infection such as sepsis has not been fully addressed. In the present study, we established Wip1 knock-out mice and applied cecal ligation and puncture (CLP) model to explore the role of Wip1 in sepsis. We also obtained human peripheral neutrophils from healthy donors and patients with sepsis to investigate the potential relation between Wip1 and neutrophils during sepsis.

## Materials and Methods

### Mice

Wip1 Knock-out (Wip1 KO) C57BL/6J mice were kindly provided by the Key Laboratory of Human Diseases Comparative Medicine (Beijing, China). WT C57BL/6J mice and CD45.1^+^ C57BL/6J mice were purchased from Model Animal Research Center of Nanjing University (Nanjing, China). All the mice were bred and maintained in the condition of specific pathogen-free. Male 8- to 10-week-old littermate mice were used for experiments. All the animal experiments were carried out following the Guidelines for the Care and Use of Laboratory Animals of Nanjing University, and were approved by the ethical review committee of Nanjing University.

### Patients

Patients were recruited from Affiliated Drum Tower Hospital of Nanjing University Medical School during May 2016 to December 2016. All patients signed the written informed consent before any study, and this study was approved by Human Subjects Institutional Committee of Drum Tower Hospital. All subjects enrolled in this study fulfilled the criteria defined by the third International Consensus Definitions for Sepsis and Septic Shock (2). Criteria for sepsis: suspected or documented infection and an acute increase of ≥2 SOFA points; criteria for septic shock: if patients with sepsis also displayed persisting hypotension requiring vasopressors to maintain MAP ≥ 65 mmHg and had a serum lactate level > 2 mmol/l (18 mg/dl) despite adequate volume resuscitation, they were selected in the septic shock group. 12 patients with sepsis and 9 patients with septic shock were enrolled within 24 h of the onset of sepsis and met the entry criteria at the time of enrollment, and blood samples were collected, which were further used for comparison with healthy male and female donors. Patients were excluded if they were <18 or >75 years age, were neutropenic (<1,000 white blood cells per microliters), were with irreversible circulatory shock, or were known to have human immunodeficiency virus infection.

### CLP Experimental Sepsis Model

Experimental CLP sepsis model were conducted as described previously (13). Briefly, mice were anesthetized and the cecum was then ligated below the ileocecal valve and punctured twice with an 18-gauge needle. Sham-operated animals (control) underwent identical laparotomy but without cecum ligation or puncture. The survival rates of animals were observed every 24 h to 7 days after surgery.

### Measurement of ALT, AST, BUN, and Cre Levels

Peripheral blood samples were acquired from mice at indicated time points and the levels of ALT, AST, BUN, and Cre were measured using commercially available automatic chemical detector (model number: HEMAVET 950FS) from Shandong Zhuoyue Company according to manufacturer’s instructions.

### Measurement of Bacterial Count in CLP Model

Peritoneal exudate and blood specimens were collected from mice under sterile conditions, and serial dilution of these samples were plated on blood-agar base plates (Trypticase Soy Agar Deeps, BD), which were incubated at 37°C overnight. Colony-forming units were recorded the next day.

### Cell Isolation

Bone marrow cells, peritoneal cavity macrophages, and peripheral white blood cells were obtained as described previously (14). For isolation of neutrophils from bone marrow cells, bone marrow cells were isolated and laid on top of a two-layer percoll (Sigma-Aldrich) gradient (72 and 65% in HBSS). Mature neutrophils were recovered at the interface of the 65–72% fractions. The cells isolated were usually more than 95% Ly6G-positive as determined by flow cytometry (Aria II; BD).

### Depletion of Neutrophils and Adoptively Transfer

To deplete neutrophils *in vivo*, 0.25 mg depleting anti-Ly6G mAb (clone 1A8; BioLegend, San Diego, CA, USA) were injected i.p. 24 h before CLP procedure. For CXCR2 inhibition studies, the CXCR2 antagonist SB225002 (10 mg/kg, Cayman Chemical, Ann Arbor, MI, USA) were injected i.p. 1 h before CLP surgery (15). For the *in vivo* recruiting experiments, neutrophils from WT or Wip1 KO were labeled with PKH or CFSE *in vitro* and were injected i.v. together (4 × 10^6^: 4 × 10^6^) into neutrophil-depleted WT-recipient mice after CLP induction. PKH or CFSE-labeled neutrophils from WT or Wip1 KO donors were quantified in recipient peritoneal cavity 3 h after CLP by flow cytometry (Aria II; BD).

### Chemotaxis Assay

Chemotaxis was performed in a 48-well microchamber (Neuro Probe) through the use of a 5-μm-pore polycarbonate membrane as previously described (16). Neutrophils (1 × 10^6^) were assayed in response to CXCL8 (30 ng/ml) or medium alone for 1 h. Neutrophils, which migrated through the membrane were counted under a light microscope on at least five randomly selected fields (17).

### Wip1 Inhibitor Treatment

In some experiments, the mice were injected i.p. with the Wip1 inhibitor (CCT007093, 2.5 mg/kg in dimethylsulfoxide, CAS 176957-55-4, Santa Cruz Biotechnology) every 12 h at 48 h before CLP procedure.

### RNA Extraction and Quantitative PCR Analysis

Total RNA was isolated with TRIzol (Invitrogen, Carlsbad, CA, USA) or E.Z.N.A.™ total RNA kit I (#R6834). Reverse transcription was performed with M-MLV superscript reverse transcriptase according to the manufacturer’s instructions. Real-time PCR was performed by using multiple kits (SYBR Premix Ex Taq™, DRR041A, Takara Bio) on CFX96 (Bio-Rad) and the target gene expression was normalized to the house keeping gene hypoxanthine phosphoribosyl transferase (HPRT). The primers used in the present study include:

Human HPRT, Forward: CAAGGATGTGGATGAGAAAGCAGACA,
Reverse: ATGATGGCGTCGGTCTGGATGTAGTC,
Human Wip1, Forward: GAAGAAACTGGCGGAATGG,
Reverse: TTGTGAGTGAGTCGAGGTCGT,
Mouse HPRT, Forward: AGTACAGCCCCAAAATGGTTAAG,
Reverse: CTTAGGCTTTGTATTTGGCTTTTC,
Mouse Wip1, Forward: CTGACTGATAGCCCTACTTACAACA,
Reverse: GAGAAGGCATTACTGCGAACA,
Mouse CXCR2, Forward: TCACAAACAGCGTCGTAGA,
Reverse: GACAGCATCTGGCAGAATAG,
Mouse CXCR1, Forward: ATGGCCGAGGCTGAATATTTC,
Reverse: TTAATAAATAGCGGTGAGAGA.

### Analysis of Phagocytic Bacteria-Killing Activity

Neutrophils and macrophages isolated from WT or Wip1 KO mice were incubated with *S. aureus* or *E. coli* (a bacterial concentration corresponding to 10^6^ CFU) in flat-bottom 96-well plates (Costa) in a total of 200 µl RPMI1640 medium at 37°C in 5% CO_2_ incubator for 3 h. For phagocytosis measurement of cells, neutrophils or macrophages were collected and stained with anti-mLy6G-FITC or anti-mF4/80-FITC (ebioscience). The percentage of Ly6G^+^ or F4/80^+^ cells with phagocytosis in the gated Ly6G^+^ or F4/80^+^ cells were analyzed by flow cytometry (Aria II, BD). For *S. aureus* or *E. coli* survival measurement, cells were lysed with 0.01% Triton X-100 in water in some wells incubated with bacterial. Surviving *S. aureus* or *E. coli* CFUs were determined by plating them on Tryticase Soy Agar.

### Oxidative Burst Assay

Respiratory burst was determined as described (18). Neutrophils isolated from bone marrow were incubated in the presence of 1 µM dihydrorhodamine (Molecular Probe, Sigma-D1054) during stimulation with PMA (40 ng/ml) (Sigma-P8139). In some experiments, neutrophils isolated from bone marrow were preincubated with Wip1 inhibitor CCT007093 (10 µM) for 1 h. Then respiratory burst was measured by oxidation of dihydrorhodamine 123 after activation with 40 ng/ml PMA.

### Statistical Analyses

The data were reported as mean ± SEM. Student’s *t* test was used to compare the differences between two groups, and one-way or two-way ANOVA analysis was used for comparison among multiple groups. Survival studies were analyzed with the log-rank test, and bacterial counts were compared by the Mann–Whitney *U-*test. *P* < 0.05 was considered statistically significant.

## Results

### Wip1 Deficiency Improves the Outcome of Sepsis

**Figure 1:**
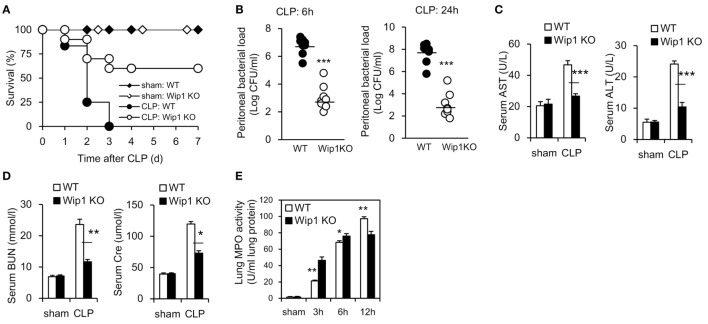
Wip1 deficiency improves the outcome of mice in CLP-induced sepsis model. Wild-type (WT) and Wip1 KO mice were given sepsis by the CLP procedure. **(A)** The animals were monitored for survival every 24 h until 7 days post-CLP. Survival rate after CLP in WT or Wip1 KO mice were expressed as a percentage (*P* < 0.05, *n* = 22). Peritoneal bacterial load **(B)** was investigated at 6 and 24 h post CLP in WT or Wip1 KO mice. The plasma levels of AST, ALT **(C)**, and BUN, Cre **(D)** were measured 24 h after surgery. Lung MPO activity **(E)** was also measured at 3, 6, and 12 h after surgery. The data (mean ± SEM) are representative of 6–8 mice per group from two independent experiments. *P < 0.05, **P < 0.01, ***P < 0.001. CLP, cecal ligation and puncture; MPO, myeloperoxidase.

Wild-type and Wip1 KO mice were subjected to lethality CLP surgery and the survival was monitored. WT mice all died by 72 h after CLP surgery as described previously (13), while Wip1 KO were highly resistant to CLP-induced lethality with more than half of the mice were still alive at day 7 (Figure 1A). We next examined whether resistance to CLP-induced sepsis in Wip1 KO mice was related to an enhanced bacterial clearance. The bacterial load in the peritoneal cavity at 6 and 24 h after CLP was significantly lower in Wip1 KO mice as compared with WT mice (Figure 1B), and similar results were also found in the bacterial load of peripheral blood (Figure S1A in Supplementary Material), indicating that Wip1 deficiency results in enhanced bacterial clearance after CLP surgery. Plasma ALT and AST levels were also significantly lower in Wip1 KO mice as compared with WT mice 12 h after CLP (Figure 1C), as well as lower plasma levels of BUN and Cre (Figure 1D). Other systemic inflammation markers including neutrophil accumulation in the lungs and cytokine levels (tumor necrosis factor α and IL-6) in the serum (14) were also found to be lower in Wip1 KO mice 12 h after CLP surgery (Figures 1D,E; Figure S1B in Supplementary Material). Taken together, all these results showed that Wip1 was involved in the process of sepsis, and Wip1 deficiency can improve the outcome of sepsis.

### Wip1 Intrinsically Controls Neutrophils to Regulate Sepsis

**Figure 2:**
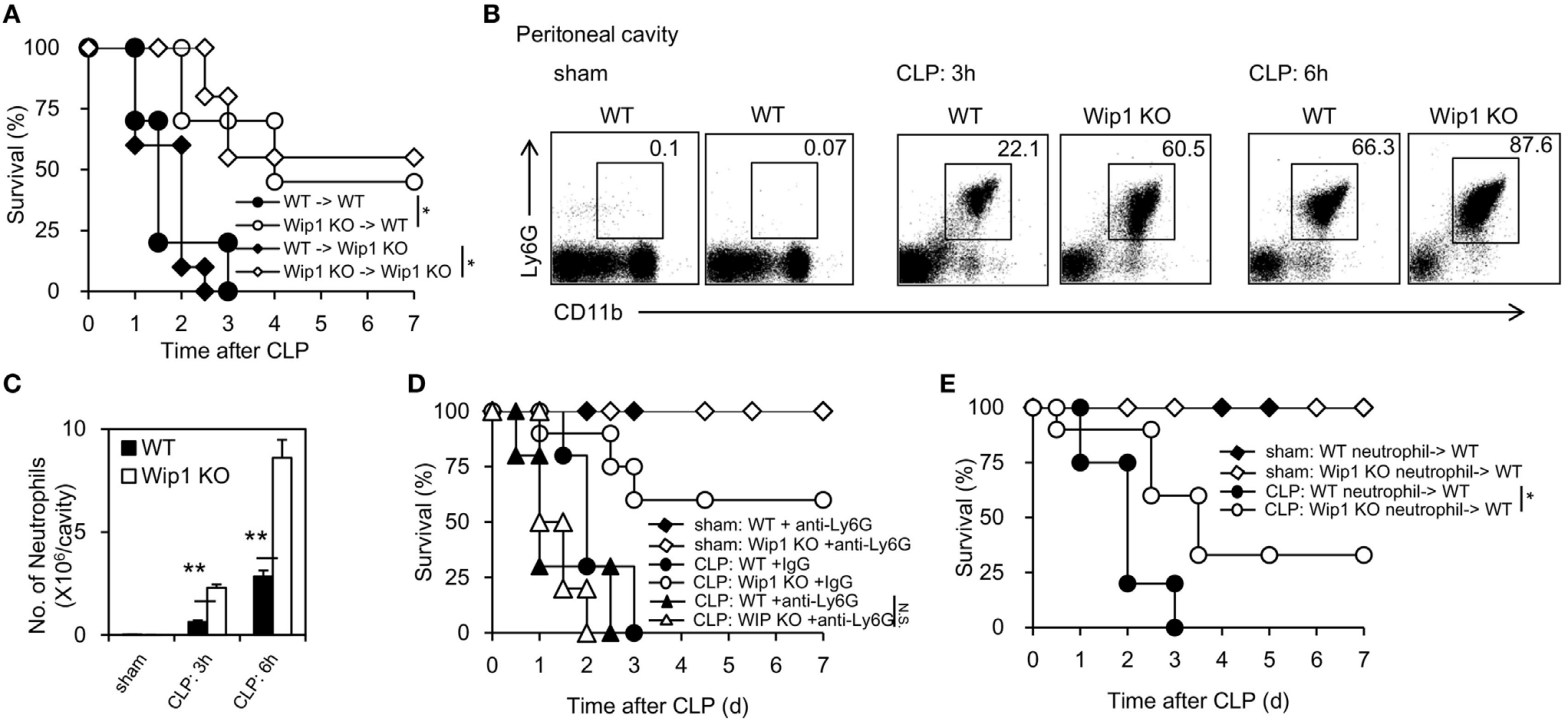
Wip1 intrinsically regulates neutrophil migration into the infection sites to improve the outcome of sepsis. Complete chimeras were generated by transferring 1–2 × 10^7^ bone marrow cells from either wild-type (WT) or Wip1 KO mice into lethally irradiated CD45.1 syngeneic mice. **(A)** Different complete chimeras were established and significantly improved survival rate was found in mice with a Wip1 deficiency in immune cells (*P* < 0.05, *n* = 20). Peritoneal exudates were harvested and analyzed for neutrophil accumulation (CD11b^+^Ly6G^+^) by flow cytometry at 3 and 6 h after cecal ligation and puncture (CLP) procedure in WT or Wip1 KO mice, and the representative flow cytometry data of neutrophil percentage **(B)** were shown between WT and Wip1 KO mice. The number **(C)** of neutrophils in the peritoneal cavity was shown (mean ± SEM, *n* = 6–8). **(D)** WT and Wip1 KO mice received either control IgG (250 µg/mouse) or anti-Ly6G Ab (250 µg/mouse) 24 h before CLP surgery. The survival was monitored for 7 days (*n* = 10). **(E)** Neutrophils (3 × 10^6^/mouse) from bone marrow of WT or Wip1 KO mice were adoptively transferred (i.v.) into neutrophil-depleted syngeneic WT mice (3 × 10^6^/mouse) after CLP surgery. All the data are composite of two independent experiments. *P < 0.05, **P < 0.01.

To explore the cellular mechanism accounting for the improved outcome of sepsis in Wip1 KO mice, complete chimeras were generated and results showed WT mice grafted with Wip1 KO BMCs displayed much more resistance to CLP-induced sepsis compared to WT mice grafted with WT BMCs (Figure 2A), indicating that Wip1 intrinsically controls hematopoietic cells to regulate sepsis. Since Wip1 is highly expressed in neutrophils (Figure S2A in Supplementary Material), we then investigated the migration of neutrophils into the peritoneal cavity, which has been found to be impaired both in animal and humans sepsis (3). Wip1 KO mice exhibited a significantly higher accumulation of neutrophils into peritoneal cavity when compared with WT mice at 3 and 6 h after CLP surgery, leading to a higher total numbers of leukocyte influx (Figures 2B,C; Figure S2B in Supplementary Material). Moreover, Wip1 KO neutrophils exhibited an increased phagocytosis to *E. coli* (Figures S3A,B in Supplementary Material) but not macrophages (Figure S2C in Supplementary Material), with an increased production of ROS (Figure S3C in Supplementary Material) despite similar formation of neutrophil extracellular trap *in vitro* (data not shown). In addition, resistance to CLP-induced sepsis in Wip1 KO mice no longer existed after depletion of neutrophils with anti-Ly6G antibody (Figure 2D; Figure S2D in Supplementary Material), and neutrophil-depleted WT mice adoptively transferred with neutrophils from Wip1 KO BMCs also exhibited partially resistance to CLP-induced sepsis (Figure 2E). Collectively, all these results demonstrated that Wip1 intrinsically drove the increased neutrophil influx to the primary infection foci during sepsis with enhanced antimicrobial function, leading to the improved sepsis outcome.

### CXCR2 Is Critical for Enhanced Neutrophil Migration into Infection Foci in Wip1 KO Mice during Sepsis

**Figure 3:**
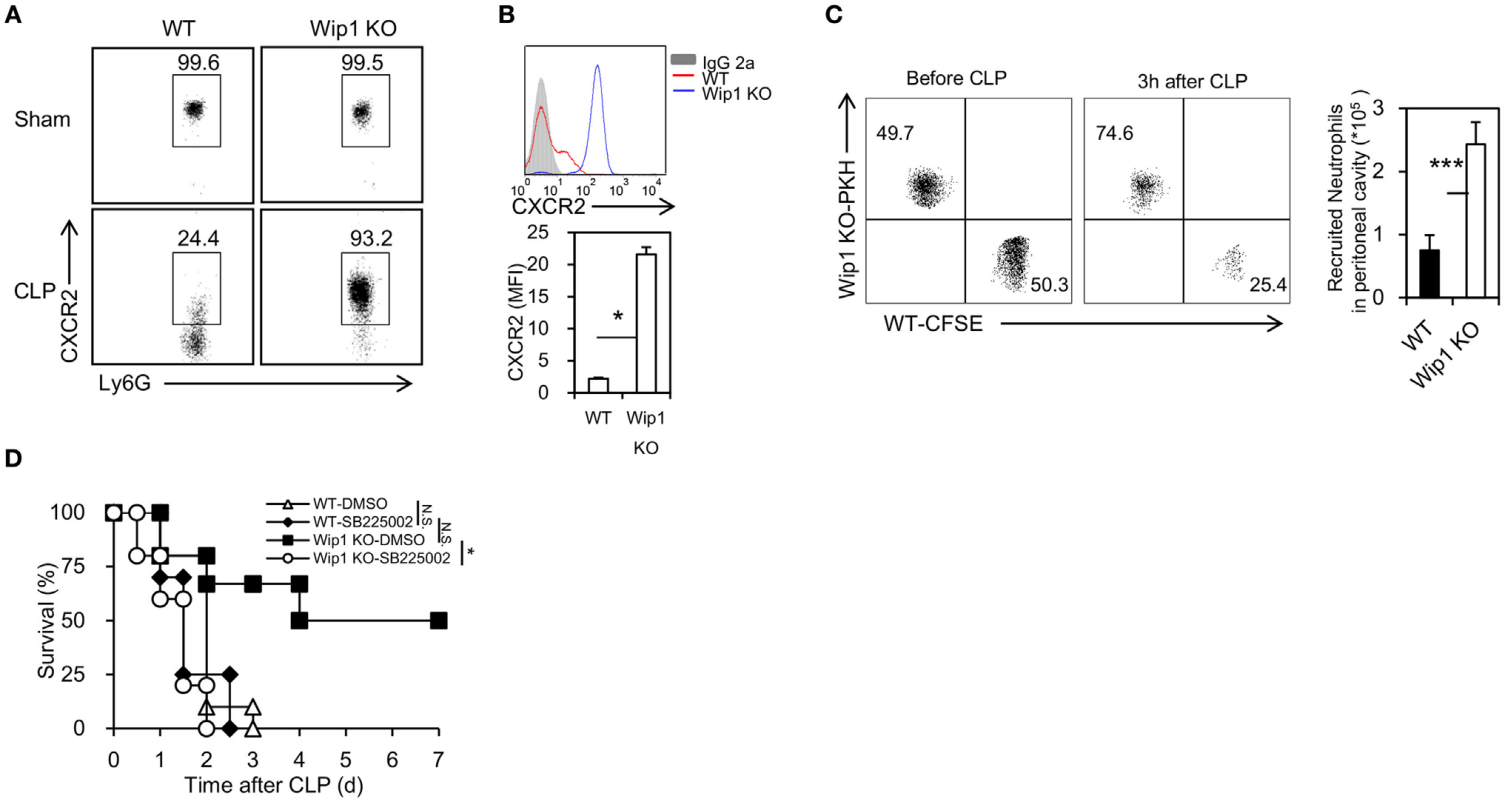
Wip1 deficiency prevents the internalization of CXCR2 on neutrophils during sepsis. The levels of CXCR2 in peripheral neutrophils from wild-type (WT) and Wip1 KO mice were measured 2 h after surgery by flow cytometry. Representative flow cytometry data of the percentage of CXCR2^+^ cells in Ly6G^+^ cells **(A)** and mean fluorescence intensity (MFI) of CXCR2 **(B)** were shown. **(C)** Sorted WT and Wip1 KO CD11b^+^Ly6G^+^ neutrophils were labeled with PKH and CFSE and were then i.v. injected together to syngeneic mice for the *in vivo* recruiting assays. **(D)** WT and Wip1 KO mice received either control DMSO or CXCR antagonist SB225002 (10 mg/kg body weight/mouse) 1 h before cecal ligation and puncture surgery. The survival was monitored for 7 days. All the data are composite of two independent experiments. *P < 0.05, ***P < 0.001, N.S., not significant.

To explore how Wip1 controls neutrophil accumulation in the peritoneal cavity, we first assessed whether the accumulation of neutrophils in the peritoneal cavity may be a result of increased release from bone marrow. Wip1 KO mice had an expanded pool of neutrophils in the periphery at 3 and 6 h after CLP surgery (Figure S4A in Supplementary Material). The recruitment of neutrophils into infection sites is mainly mediated by CXCR2 functioning with CXCL1 and CXCL2. The expression of CXCR2 in peripheral blood neutrophils from Wip1 KO mice remained high at 2 h after CLP surgery (Figures 3A,B), and no significant difference was found in serum CXCL1 and CXCL2 levels, as well as levels of CXCR2 mRNA expression in neutrophils between WT and Wip1 KO mice after CLP (Figures S4B,C in Supplementary Material). The mRNA expression level of CXCR1, which is also expressed on mice neutrophils and has been identified to contribute to Candida clearance recently (19), was also identical between Wip1 KO and WT neutrophils after CLP (Figure S4C in Supplementary Material). Moreover, neutrophils isolated from Wip1 KO mice also displayed increased recruitment into the peritoneal cavity after CLP surgery when adoptively transferred with neutrophils from WT mice into neutrophil-depleted WT mice (Figure 3C). Pharmacological inhibition of CXCR2 with SB225002 also resulted in the abrogation of the prolonged survival in Wip1 KO mice after CLP-induced sepsis (Figure 3D). Our previous results showed that Wip1 deficiency can lead to the increased phosphorylation of p38, which can bind directly to CXCR2, thus decreasing the internalization of CXCR2 (20), all these results suggestd that Wip1 negatively regulated neutrophil migration into infection sites during sepsis through preventing CXCR2 internalization.

### Pharmacological Inhibition of Wip1 *In Vivo* Improves the Outcome of Sepsis

**Figure 4:**
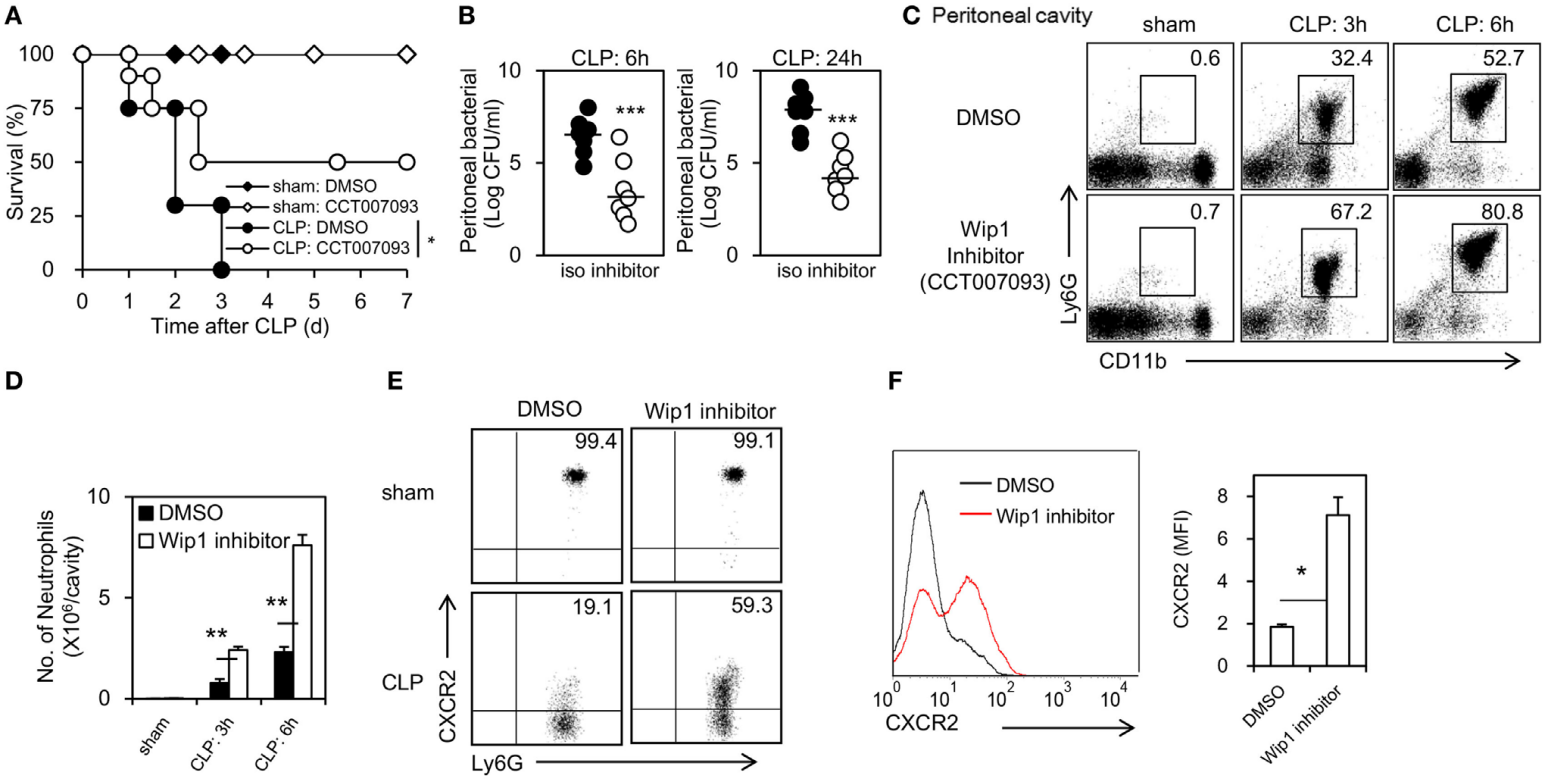
Pharmacological inhibition of Wip1 improves the outcome of sepsis through mastering neutrophil migration into the infection sites. Wild-type mice were injected i.p. with the Wip1 inhibitor (CCT007093, 2.5 mg/kg in dimethylsulfoxide, CAS 176957-55-4, Santa Cruz Biotechnology) every 12 h at 48 h before cecal ligation and puncture (CLP) procedure. **(A)** The survival rate was monitored for 7 days and was shown. **(B)** Peritoneal bacterial load was compared between mice treated with DMSO or CCT007093 (Wip1 inhibitor) at 6 and 24 h after CLP. Peritoneal exudates were harvested and analyzed for neutrophil accumulation (CD11b^+^Ly6G^+^) by flow cytometry at 3 and 6 h after CLP procedure in mice treated with either DMSO or Wip1 inhibitor CCT007093. Representative flow cytometry data of the percentage of CD11b^+^Ly6G^+^ neutrophils **(C)** was shown. The numbers of neutrophils harvested **(D)** were compared between mice treated with either DMSO or Wip1 inhibitor CCT007093 (mean ± SEM, *n* = 6–8). The percent of CXCR2^+^ cells **(E)** and mean fluorescence intensity (MFI) of CXCR2 **(F)** in Ly6G^+^ neutrophils were also shown. MFI are expressed as mean ± SEM (*n* = 6–8). All the data are composite of two independent experiments. *P < 0.05, **P < 0.01, ***P < 0.001. iso, isotype.

To test whether pharmacological inhibition of Wip1 can improve the outcome of sepsis, CCT007093, a specific inhibitor of Wip1, was used 48 h before CLP surgery. Pharmacological inhibition of Wip1 with CCT007093 significantly increased the survival of WT mice with less systemic inflammation and organ damage (Figure 4A; Figures S5A–F in Supplementary Material). The bacterial count in the peritoneal cavity of WT mice treated with CCT007093 was also significantly lower than that treated with DMSO at 6 and 24 h after CLP surgery (Figure 4B). Moreover, mice treated with CCT007093 exhibited a significantly increased recruitment of neutrophils into the peritoneal cavity (Figures 4C,D; Figure S6A in Supplementary Material) with decreased internalization of CXCR2 (Figures 4E,F), despite a slightly enhanced pool of neutrophil in the peripheral (Figure S6B in Supplementary Material). It is also notable that inhibition of Wip1 with CCT007093 can increase ROS production and phagocytosis to *E. coli* (Figures S6C,D in Supplementary Material). Taken together, these results demonstrated that pharmacological inhibition of Wip1 can also improve the outcome of CLP-induced sepsis.

### Correlation between CXCR2 and Wip1 in Human Neutrophils

**Figure 5:**
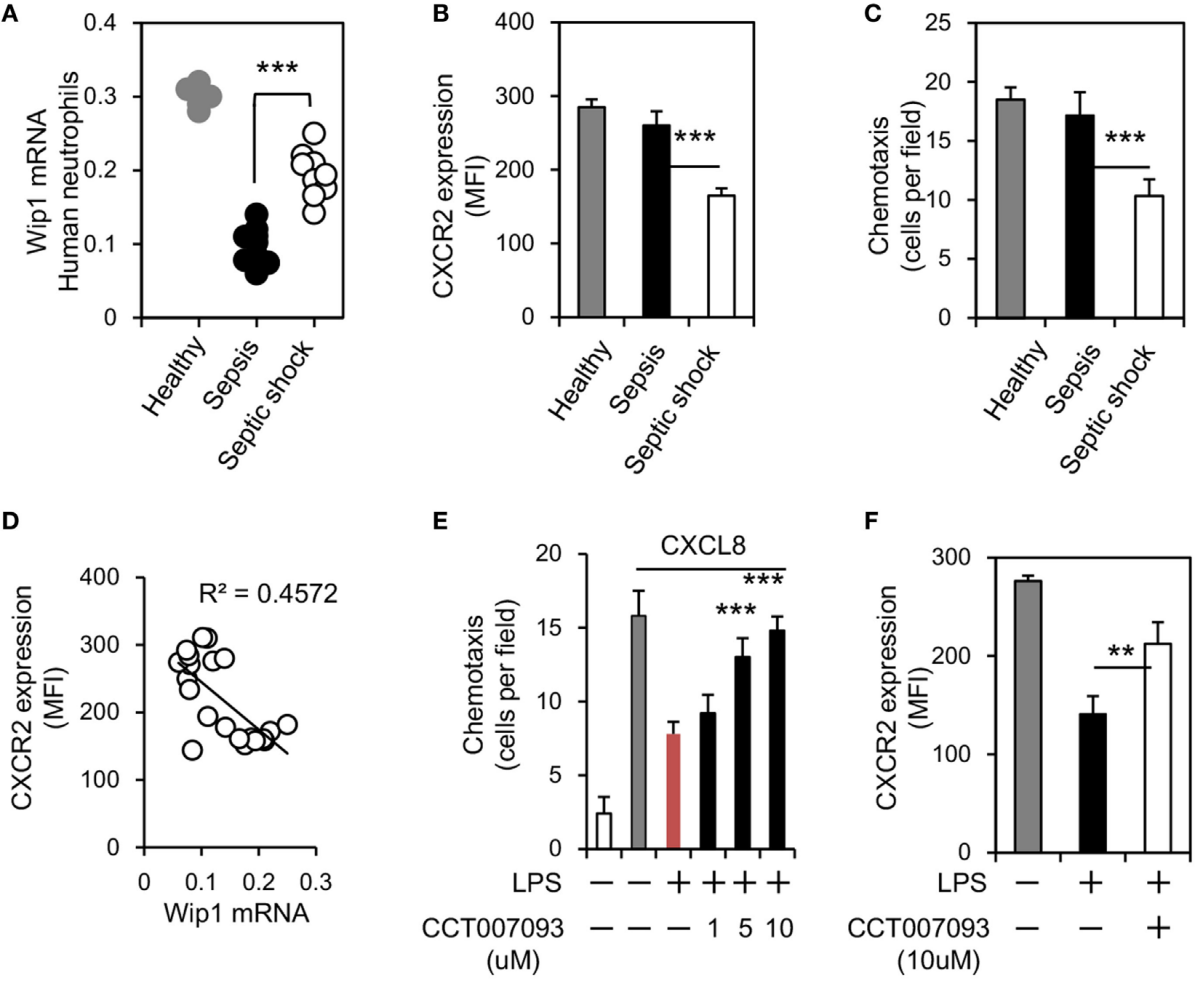
Wip1 is negatively correlated to CXCR2 expression in human neutrophils during sepsis. Human neutrophils from healthy donors, patients with sepsis, and patients with septic shock were purified from peripheral blood. **(A)** The expressions of Wip1 mRNA between human neutrophils of healthy donors, patients with sepsis, and patients with septic shock were shown (6 healthy donors, 12 patients with sepsis, and 9 patients with septic shock). **(B)** The mean fluorescence intensity (MFI) of CXCR2 in human neutrophils was compared (mean ± SEM). **(C)** Neutrophils isolated were also examined for chemotaxis (toward CXCL8), *n* = 5–9 donors per group. **(D)** The negative correlation between Wip1 and CXCR2 in neutrophils from individuals was shown. **(E)** Chemotaxis toward CXCL8 of purified peripheral blood neutrophils from healthy donors in the presence or absence of LPS (1 µg/ml) and graded concentration of Wip1 inhibitor CCT007093 were shown. *n* = 5 donors, ***P < 0.001 versus cultures without Wip1 inhibitor CCT007093. Data are mean ± SEM. **(F)** The MFI of CXCR2 in purified peripheral blood neutrophils from healthy donors were shown in the presence or absence of LPS and Wip1inhibitor CCT007093 (10 µM) (mean ± SEM, *n* = 5–9 donors per group). **P < 0.01, ***P < 0.001.

To address whether Wip1 can regulate human neutrophil migration during sepsis, we isolated neutrophils from healthy donors, patients with sepsis, or septic shock. We found Wip1 mRNA levels were both downregulated in neutrophils of patients with sepsis or septic shock, while the level of Wip1 in neutrophils of patients with septic shock was significantly higher than that with sepsis (Figure 5A). Since CXCR2 expression on neutrophils of patients with septic shock were significantly lower than those of patients with sepsis and healthy donors (Figure 5B) with an impaired migration capacity *in vitro* (Figure 5C), we then examined the possible correlation between CXCR2 expression and Wip1 in human neutrophils of patients with sepsis. Strikingly, a negative correlation between CXCR2 expression and Wip1 was identified in our patients (Figure 5D), indicating a critical role of Wip1 in regulating CXCR2 expression on human neutrophil during sepsis. Based on these results, we then explored the possibility of Wip1 inhibitor treatment in reversing the downregulated expression of CXCR2 on human neutrophils found during sepsis *in vitro*. LPS is the major component of the outermost membrane of Gram-negative bacteria and has been implicated in the clinical syndrome of Gram-negative bacterial septic shock such as severe abdominal infection (21). Treatment with LPS inhibited human neutrophil chemotaxis to CXCL8 stimulation, and this was reversed by Wip1 inhibitor CCT007093 treatment *in vitro* with maintained expression of CXCR2 on neutrophils (Figures 5E,F). Thus, our results demonstrated that Wip1 also played a critical role in human neutrophil migration during sepsis.

## Discussion

Sepsis is a complex pathophysiological process caused by dysregulated host response to infection (3). Neutrophil plays a critical role in innate immune response to infection and participates a lot both in the early and late phase of sepsis (22). Based on our previous works on defining the critical role of Wip1 in neutrophil development and immune function, we employed Wip1 KO mice and demonstrated the protection against CLP-induced sepsis in Wip1 KO mice was fully dependent on Wip1-deficient neutrophils. The paralysis of neutrophils observed during sepsis has been related to low cell surface expression of CXCR2, which is caused by increased internalization but not by the reduced mRNA levels (14, 23). In accordance with other studies, we also observed downregulated expression of CXCR2 on neutrophils from WT mice after CLP surgery, but the expression of CXCR2 on Wip1 KO neutrophils maintained high after CLP surgery. It is notable that other steps in neutrophil recruitment such as mobilization, rolling, and adhesion are also impaired during sepsis (3). Although we did not explore all the steps involved, we did find an enhanced pool of neutrophils in peripheral blood, which can be a result of the increased neutrophil mobilization in Wip1 KO mice as described by us previously (24). Two CXC chemokine receptors, CXCR1 and CXCR2, are expressed on neutrophils. CXCR1 binds with high affinity to IL-8, but binds with low affinity to ENA-78, neutrophil-activating peptide-2, and GRO-α, GRO-β, GRO-γ, whereas CXCR2 binds all these chemokines with high affinity (25). CXCR1 has been shown to be downregulated in human neutrophils during bacterial infection (26), whereas in septic patients with organ dysfunction and septic shock, CXCR1 expression was preserved and CXCR2 expression was significantly downregulated (25, 27). Due to the difficulty in identifying mouse CXCR1 (28), the role of CXCR1 on mouse neutrophils has also been controversial. With the use of CXCR1 KO mice, Swamydas et al. recently demonstrated CXCR1 on mouse neutrophils promoted Candida clearance through mediating neutrophil degranulation and fungal killing (19). To address whether CXCR1 expression was involved in our model, we also assessed the mRNA expression level of CXCR1 on neutrophils after CLP. We found the mRNA expressions of CXCR1 on neutrophils between Wip1 KO and WT mice were identical. Since pharmacological inhibition of CXCR2 resulted in the abrogation of the prolonged survival in Wip1 KO mice, Wip1 inhibition-mediated prevention of CXCR2 internalization is the key event for the improved outcome of sepsis in Wip1 KO mice. Numerous studies have identified the mechanisms accounting for the internalization of CXCR2, such as increased expression of G protein-coupled receptor kinase-2 by toll-like receptor-2 and TLR4 activation (16, 17) and mitochondrial damage-associated molecular patterns (29). However, none of them have been applied to clinical practice, possibly due to the cost of impaired neutrophil bactericidal activity such as pathogen recognition (14).

The migration of neutrophils into infection sites is important during sepsis as mentioned above, while appropriate antimicrobial activity exerted by neutrophils is also critical. Neutrophils displayed less impaired migration capacity during sepsis when compared to those during septic shock and sepsis with organ dysfunction (3, 27, 30, 31), but the primary infection still cannot be fully controlled. Evidence that increased neutrophils during non-inflammatory condition did not lead to tissue damage (32) and neutrophils recruited to organs far from infection induced organ damage during sepsis (30, 33, 34) further confirmed the notion. Increased life span of neutrophils during sepsis (3) is also suggested to contribute to tissue damage. Therefore, despite the impaired migration of neutrophils during sepsis, simple modification of neutrophil migration into infection site is not enough for sepsis treatment. How to reverse the dysregulated antimicrobial response of neutrophils recruited into infection sites is also critical for sepsis treatment (3). The antimicrobial activity of neutrophils to pathogens includes phagocytosis, ROS production, and NET formatin. We found Wip1 deficiency not only decreased the internalization of CXCR2 on neutrophils but also led to an enhanced ROS production and bactericidal activity *in vivo* and *in vitro*. Short-term use of Wip1 inhibitor can also increase ROS production and phagocytosis to *E. coli* in WT neutrophils. Thus, Wip1 inhibition not only promoted neutrophil migration but also enhanced neutrophil antimicrobial function during sepsis. The formation of NETs between WT and Wip1 KO mice were identical. Interestingly, neutrophils from Wip1 KO mice displayed an increased phagocytosis to *E. coli* but not to *S. aureus in vitro*, and the percentage of survival for *E. coli* cultured with Wip1 KO neutrophils was also significantly decreased when compared to that with WT neutrophils. Reasons for the inconsistency of *in vitro* results are still not clear. Nevertheless, our previous results also showed that Wip1 KO mice displayed an increased resistance to *S. aureus in vivo* (20). Since CLP model is a multiple microbial infection, control of both Gram-positive bacteria and Gram-negative bacteria is necessary for survival. Thus, increased infiltration of neutrophils in peritoneal cavity and an increased bactericidal activity both contributed to an improved survival in Wip1 KO mice.

Previous results have shown that Wip1 is recognized as an oncotarget (7), and modulating its activity such as the use of Wip1 inhibitor has been proven to reduce human tumor cell line viability (35, 36). Recent studies also showed the benefits with the use of Wip1 inhibitor after liver resection (37). Our study further suggested the potential application of Wip1 inhibitor in sepsis treatment. We demonstrated that pharmacological inhibition of Wip1 with its inhibitor CCT007093 can significantly improve the outcome of sepsis with increased accumulation of neutrophils in the peritoneal cavity mediated by decreased internalization of CXCR2. Several families of pattern-recognition receptors, including toll-like receptors (TLRs), are essential in the sensing of conserved microbial molecular motifs (38). Detection of microbial products such LPS by TLRs expressed on neutrophils not only activates a complex cellular response but also leads to the internalization of CXCR2 on mice and human neutrophils. Previous result has shown that IL-33 treatment can reverse the downregulated CXCR2 expression induced by LPS treatment and increase chemotaxis to CXCL-8 in human neutrophils. We also found that Wip1 inhibitor can exhibit similar effects on human neutrophils, which further suggested the potential use of Wip1 inhibitor in clinical trials. Interestingly, we did not find significantly enhanced pool of neutrophils (seen in Wip1 KO mice) in the peripheral blood of WT mice treated with CCT007093, which may partly explained that the short-term use of Wip1 inhibitor mainly affected the function of neutrophils but not their mobilization in the bone marrow. In general, our study identified a critical role of Wip1 in CLP-induced sepsis and suggested a new therapeutic strategy in the clinical setting for poly-microbial sepsis.

## Ethics Statement

All the animal experiments were carried out following the Guidelines for the Care and Use of Laboratory Animals of Nanjing University and were approved by the ethical review committee of Nanjing University. All patients signed the written informed consent before any study, and this study was approved by Human Subjects Institutional Committee of Drum Tower Hospital.

## Author Contributions

J-FD and YZ conceived and designed the experiments; X-FS, YZ, and KC performed the experiments; W-XG collected the human samples and did the experiments with human neutrophils; XL and QZ analyzed the data; J-FD and Y-TD contributed reagents/materials/analysis tools; X-FS wrote the paper.

## Conflict of Interest Statement

The authors declare that the research was conducted in the absence of any commercial or financial relationships that could be construed as a potential conflict of interest.
