# Supplementary figures and images for: Wip1 Deficiency Promotes Neutrophil Recruitment to the Infection Site and Improves Sepsis Outcome
Source: Front Immunol. 2017 Aug 22;8:1023. doi: 10.3389/fimmu.2017.01023 (PMC5572246; doi:10.3389/fimmu.2017.01023)

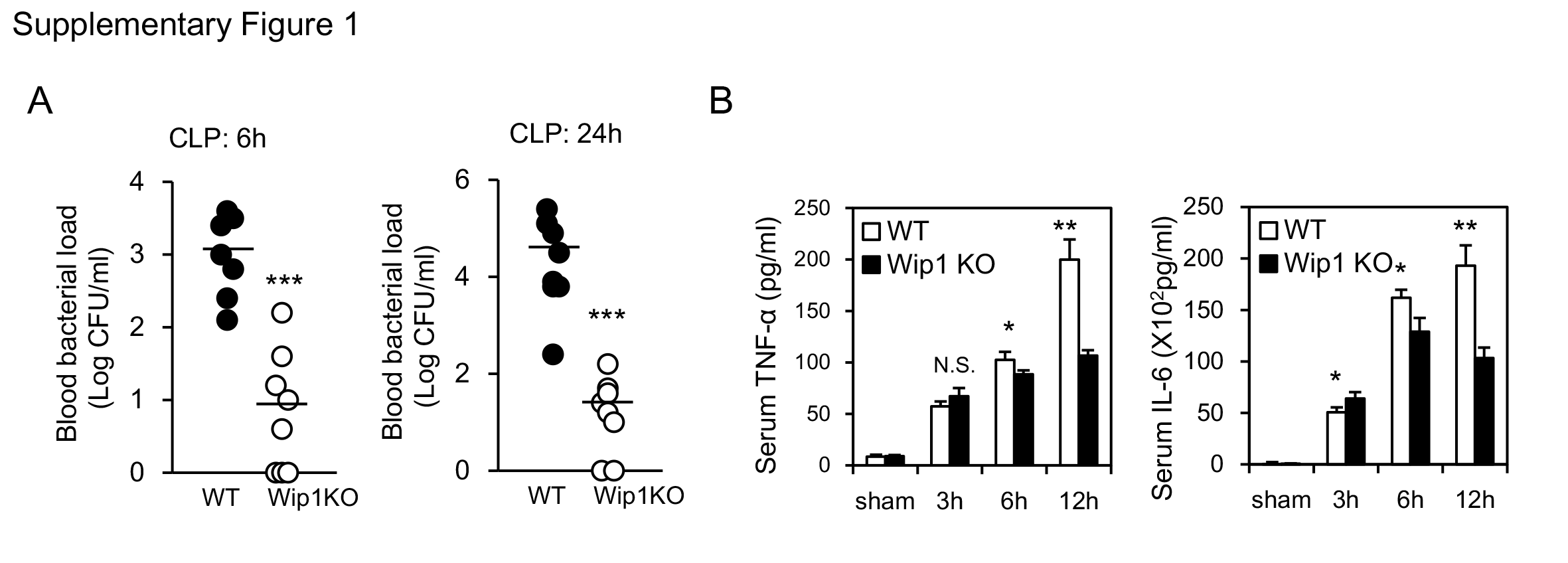

Supplement: Supplementary file 1 [file Image_1.TIF]

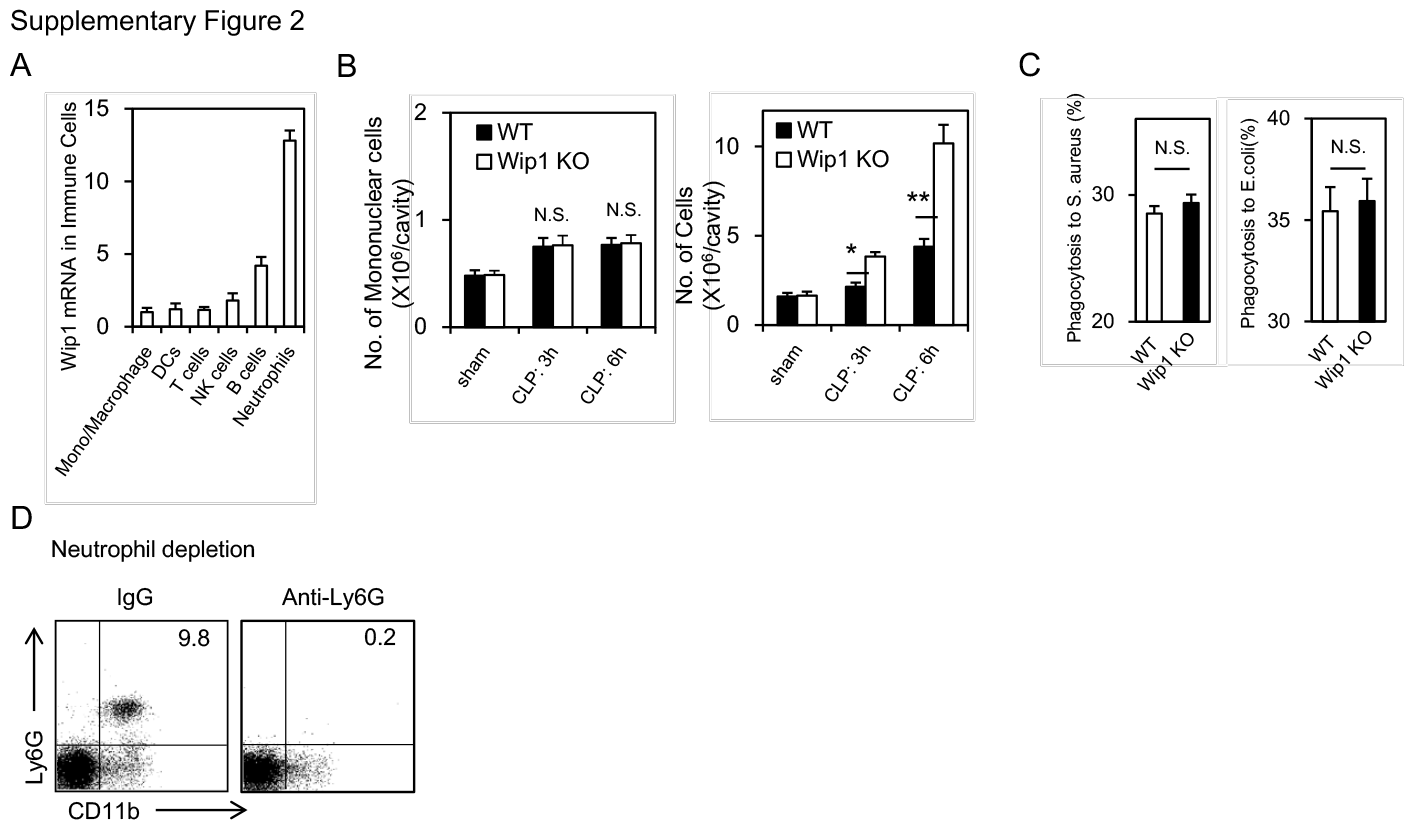

Supplement: Supplementary file 2 [file Image_2.tif]

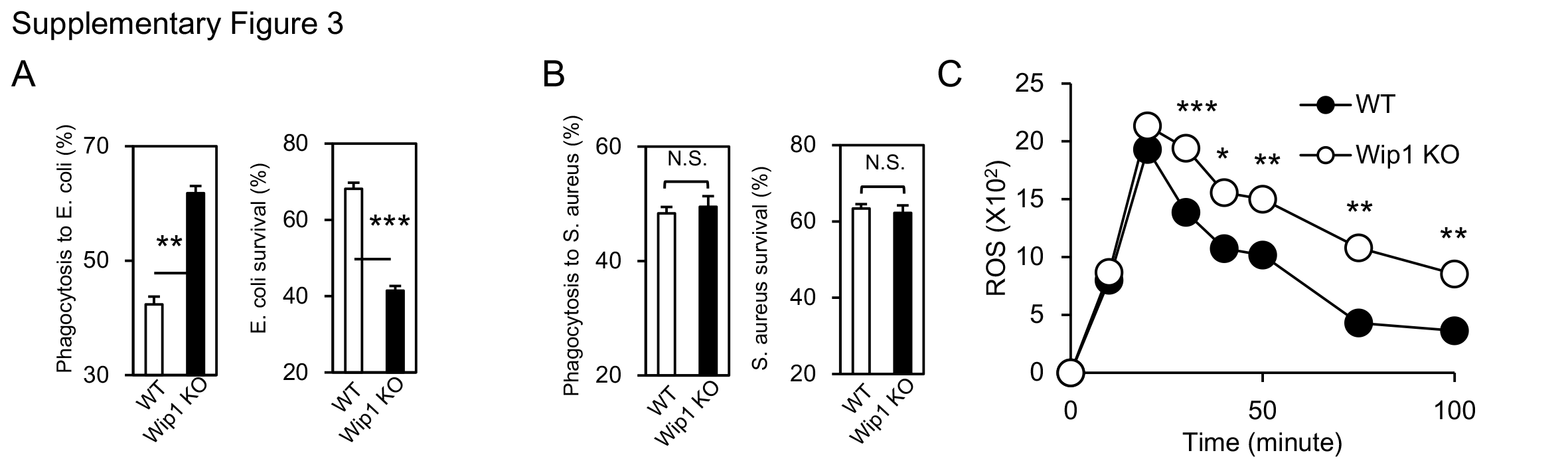

Supplement: Supplementary file 3 [file Image_3.TIF]

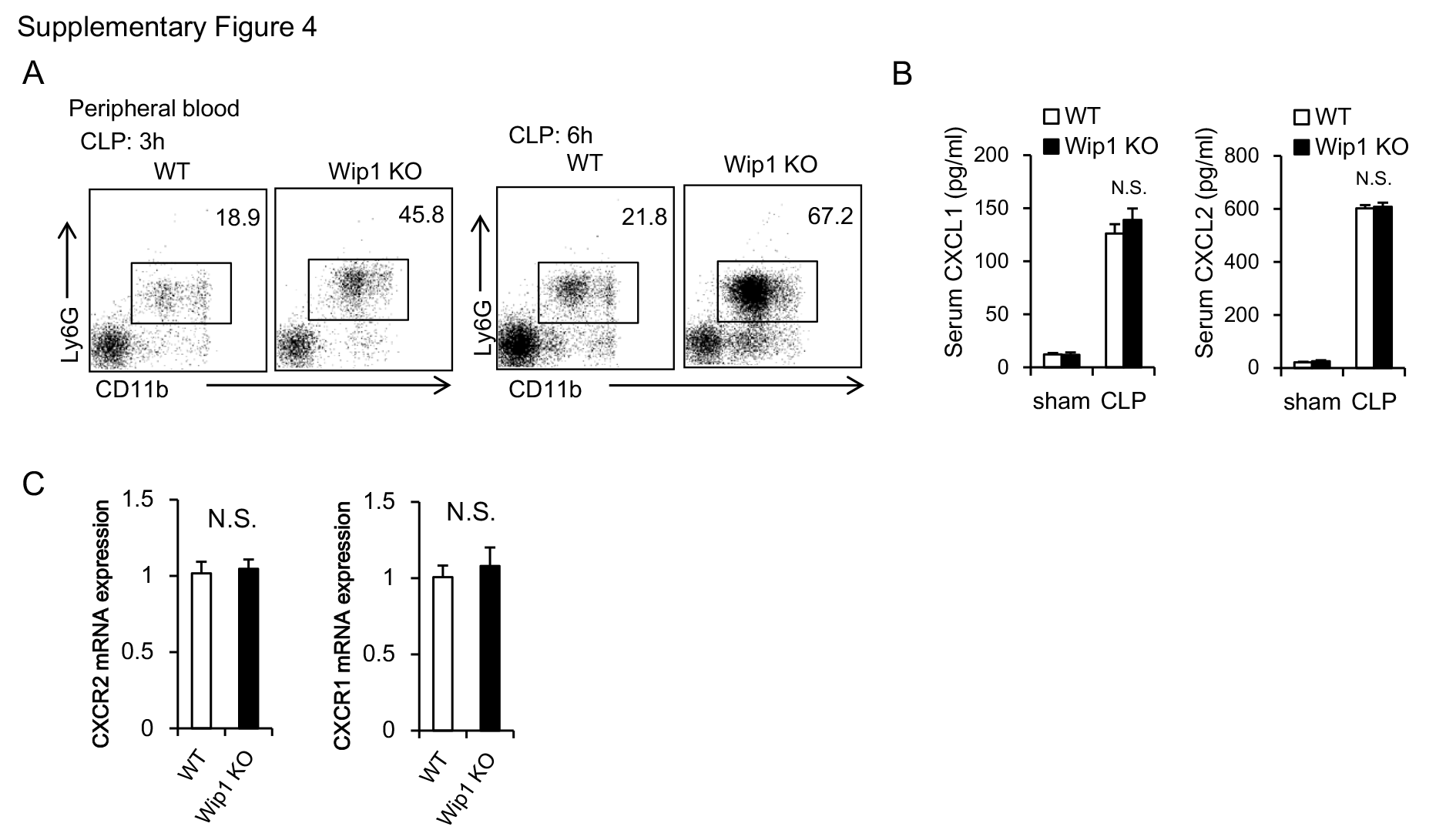

Supplement: Supplementary file 4 [file Image_4.TIF]

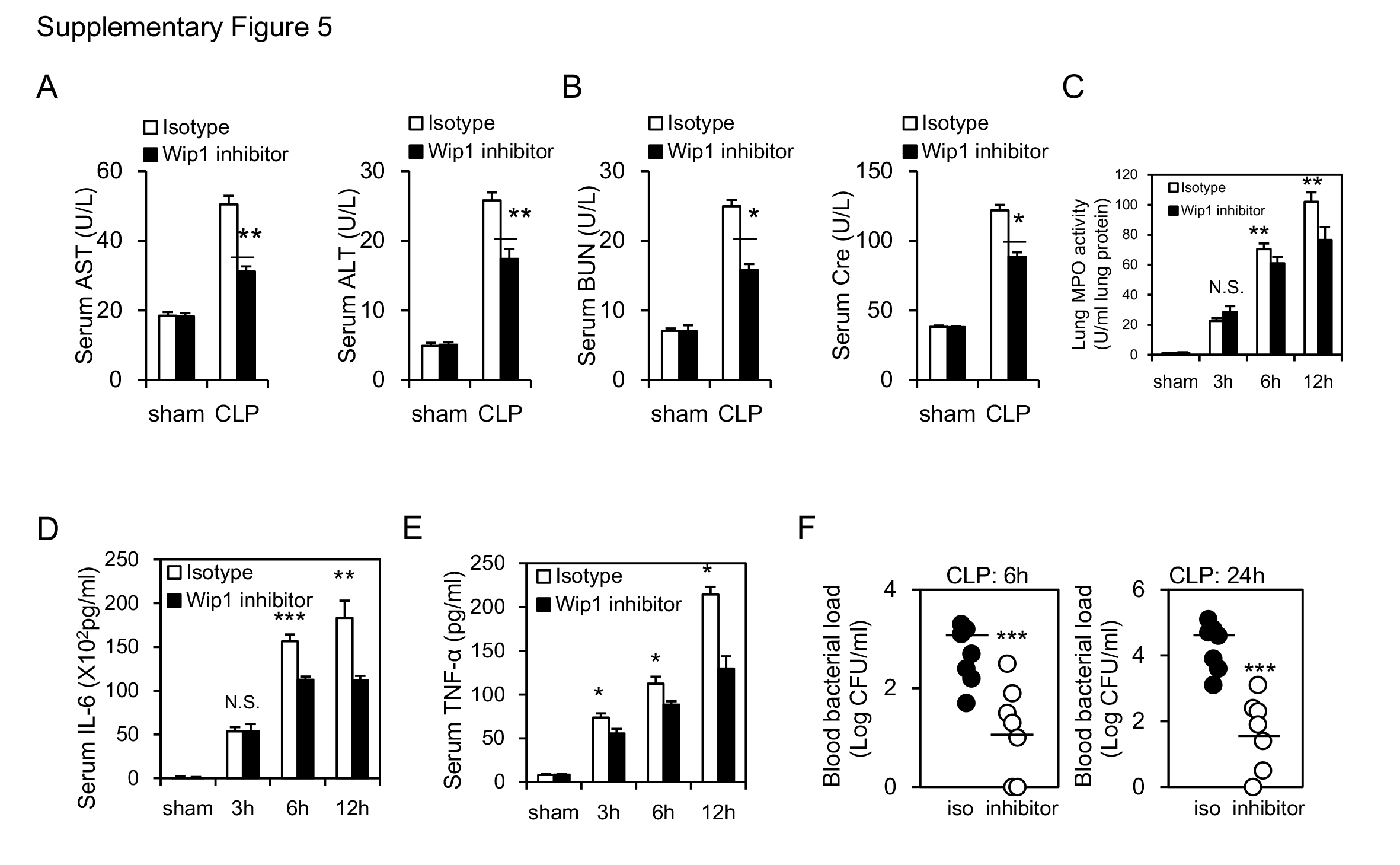

Supplement: Supplementary file 5 [file Image_5.TIF]

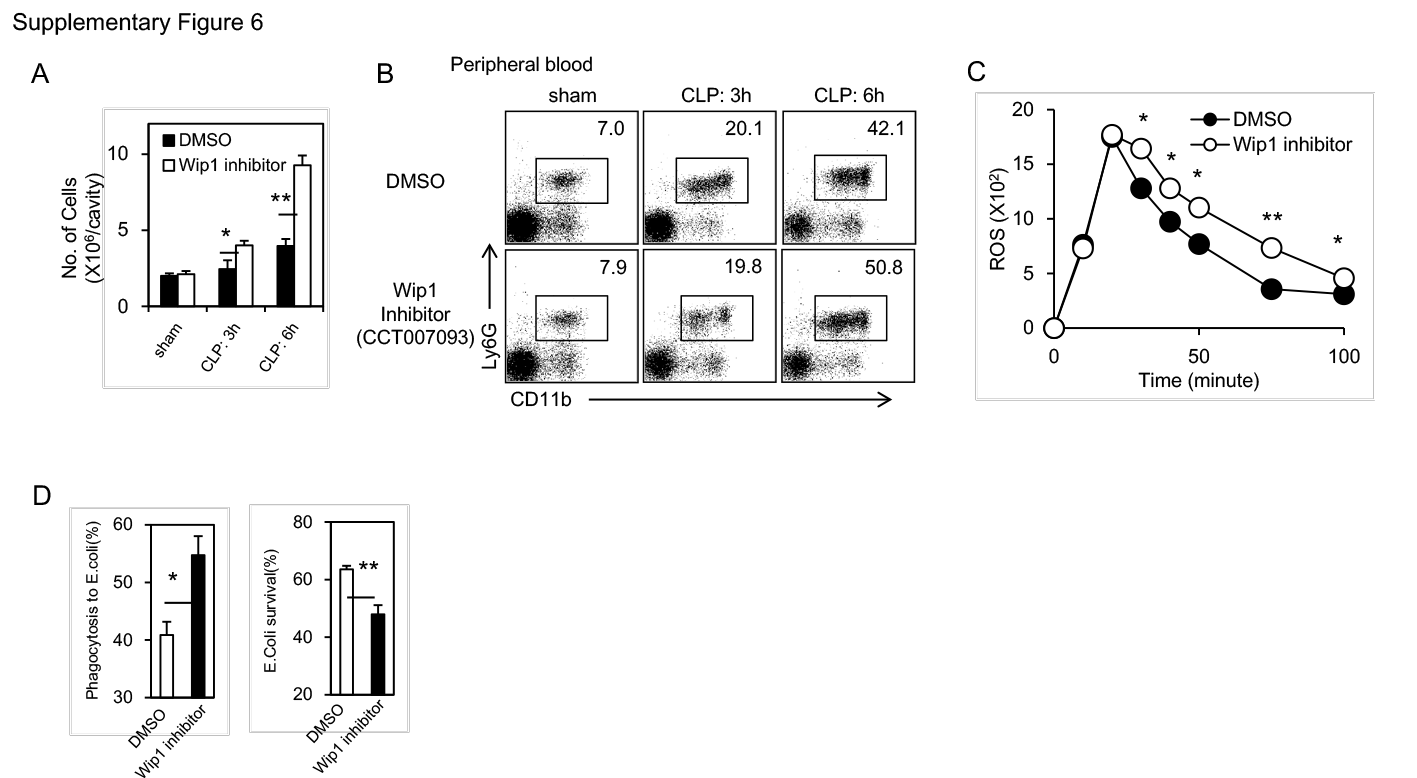

Supplement: Supplementary file 6 [file Image_6.tif]
